# Benefits and challenges associated with implementation and ongoing use of automated dispensing cabinet for medicines: A scoping review

**DOI:** 10.1016/j.rcsop.2025.100599

**Published:** 2025-04-01

**Authors:** Yoo Young Jung, Áine Walsh, Jig Patel, Kit Lai

**Affiliations:** a King's College University, Stamford St, London SE1 9NH, UK; b Pharmacy department, Level 1 Cheyne Wing, King's College Hospital NHS Foundation Trust, SE5 9RS, London, UK; c Department of Haematological Medicine, King's College Hospital, SE5 9RS, London, UK; d Institute of Pharmaceutical Science, King's College London, SE5 9RS, London, UK

**Keywords:** Automated dispensing cabinets, ADC implementation, Hospital pharmacy, Medication management, Healthcare technology, Pharmacy automation, ADC benefits, ADC challenges, Patient safety, Hospital efficiency, Controlled drugs, Healthcare innovation, Staff efficiency, Cost saving, Productivity, Inventory management, Override, Medication errors, Workflow optimization, Nursing workflows

## Abstract

When deciding whether to adopt a digital healthcare technology, there is a need to fully grasp the benefits as well as understand any potential challenges from the outset, to enable appropriate mitigations to be considered as part of implementation plans. Automated dispensing cabinets (ADCs) have been increasingly used in hospitals as a means of streamlining medicines use workflows, facilitating medicine management, saving costs, and improving patient safety. Manufacturers commonly reference the benefits of ADCs but rarely outline the challenges. It is important that senior leaders in healthcare understand both the benefits and challenges of ADCs prior to implementation, to ensure the technology is implemented in areas where the benefits can be most maximally achieved and the challenges mitigated in so far as possible. A scoping review methodology was used to map existing literature focussing on the benefits and challenges of ADC use with medicines. Following a preliminary search to identify key terms, extensive literature searches were conducted in Medline, PubMed, CINAHL, Embase, Global Health, and Web of Science. Among 234 articles identified from the search, 54 articles were included for full data extraction. Extracted information included publication date and origin, study aims & objectives, study setting, medication distribution model, technology infrastructure, overarching category, area of focus, and key findings. The findings were discussed in terms of implications for broad trends and future research directions. Although results indicate that there is an abundance of published literature on benefits and challenges associated with ADC use with medicines, there are only a handful of UK studies. Differences in settings, distribution models, workflows and technology infrastructure limit the overall ability to generalise findings. Further UK-based studies carried out in different settings with varying levels of technological infrastructure is imperative to not only track the impact of ADCs but also to inform practice to ensure the continued delivery of benefits. Further studies focusing particularly on the impact of stock optimisation and the management of CDs (Controlled drugs) would be key areas of focus.

## 1 Introduction

Advancements in technology solutions are revolutionising healthcare. The automation of processes within hospital settings to improve efficiency and patient safety is becoming increasingly common. Automated dispensing cabinets (ADCs) are one form of technology that have been in use in healthcare settings since the 1990s.^1^ ADCs are computerised medication storage cabinets used in healthcare settings to facilitate the storage of medications near the point of care. ADCs restrict and track user access as well as improve the management and tracking of drug distribution and drug inventory. The benefits of ADCs have broadly been described to include the streamlining of medication management, optimisation of workflow efficiencies, cost efficiencies, and improving the quality of care provided to patients.^1^^,^^2^ In turn, these enhancements enable the shift of focus from manual inventory management tasks to clinical patient facing tasks which was the overarching aim of the Carter Report.^3^

The assessment of the effectiveness of digital technologies can be difficult. Not least, the ability to seamlessly interface with existing solutions. In addition, assessing the benefits of ADCs in isolation can be challenging when more than one system co-exists. It is therefore useful to evaluate studies where ADCs are used in conjunction with a range of technologies in order to determine what works best.

Pharmacy departments are frequently approached in organisations to lead on implementation of ADCs. When deciding whether to adopt a digital healthcare technology, it is important for organisations to fully grasp the benefits of ADCs to define and assess the goals of implementation locally ahead of deployment. The importance of a clear vision from the outset of any change management project is widely recognised as one of the factors influencing a successful outcome.^4^ A focus on benefits realisation is also imperative, not only for the end users of the system but also for business managers to ensure the appropriate resource is put in place to manage the systems. Potential challenges to ADC implementation also need to be addressed from the outset to enable any mitigations to be devised as part of successful project plans. ADC implementation costs are no doubt a determining factor for whether these solutions are approved and funded for use, therefore understanding the implications of ADC implementation in terms of return on investment could be an important consideration in making the decision to introduce ADCs or not. It is imperative that literature is available to endorse the potential opportunities to support organisations to develop robust business cases for investment. Specifically, at the time of writing, the authors were not aware of any overarching synthesis of literature on ADCs that can be referenced.

Evaluating digital healthcare technology can be challenging. Evidence gathering is time-consuming. This may be the reason why, to date, a mapping of the landscape of research regarding benefits and challenges of ADC use with medicines is yet to be fully conducted. A scoping review is a process of mapping key concepts, the main sources, and types of evidence available as well as gaps in a research area especially where an area is complex or not previously reviewed in a comprehensive manner. The objective of this study was to explore the nature and content of published literature focussing on benefits and challenges of ADC use with medicines, identifying possible gaps in the published literature in this field. The aim of the scoping review is to allow organisations to better understand the benefits and challenges with ADCs in order to make implementation easier and to maximise the systems potential benefits.

## 2 Method

### 2.1 Overview

The use of ADCs for medicines, with associated benefits and/or limitations, was explored using a scoping review guided by the methodology of Arksey and O'Malley.^5^ An iterative approach was undertaken to develop the research question. In the first instance, the initial aim was to map the extent, range, and nature of ADC publications and to identify possible gaps among the literature in the field. This paper adheres to the Preferred Reporting Items for Systematic Reviews and Meta-Analyses (PRISMA) extension for scoping reviews.^6^

### 2.2 Search strategy

A preliminary search was conducted to identify keywords to inform the full search strategy and relevant literature. Different nomenclature for ADCs were identified including Automated Dispensing System (ADS) and Automated Distribution Device (ADD). Other key terms to specify the setting to hospital wards were compiled: inpatient, hospital, and ward.

An extensive literature search was then conducted across six databases between the 9th and 13th May 2024: Medline, Embase, PubMed, CINAHL, Global Health, and Web of Science. Inclusion criteria of the database search query was composed of two search concepts - the intervention (ADC) and the setting (hospital). Language, year and country of publication filters were not applied. Additional filters were then applied through the screening processes. Detailed search strategy and terminology have been collated in Supplementary Material 1. Additional publications were also included from external sources following reviewer feedback.

### 2.3 Inclusion & exclusion criteria

Publications were deemed eligible for inclusion if they discussed the use of medicine ADCs across the inpatient setting of hospitals, including but not limited to controlled drugs (CDs). Publications were included if authors studied the benefits or limitations of ADC use where ADCs were the primary intervention being studied. Papers were not excluded based on study type. No date restrictions were applied.

Publications were excluded if full texts were in a language other than English or if they focused on settings outside of inpatients. Papers outlining general use of ADCs were also excluded from further review where perceived benefits or challenges were not a focus. Detailed inclusion and exclusion criteria have been collated in Table 1.

**Table 1:** Description of inclusion and exclusion criteria.

	Inclusion criteria	Exclusion criteria
**Population (P)**	Studies in hospital setting, including dispensary or central pharmacy	Studies in outpatient, community settings, and operating theatres
**Intervention (I)**	Studies with ADC usage for all medicines, including CDs Studies with ADCs as the primary intervention e.g. Closed-Loop Electronic Prescribing	Studies where ADCs were not included Studies with non-ADC technologies as the primary intervention - for example dispensing robots, unit dose drug distribution (UDDS), carousel dispensing technology (CDT) Studies with ADC usage where products other than medicines are stored
**Comparison (C)**	–	-
**Outcomes (O)**	Studies that discussed perceived benefits and or challenges of ADC use for medicines	Studies where ADCs are discussed generally without specific reference to benefits and or challenges
**Study Design (S)**	All study designs included	-
**Time (T)**	No restrictions applied	-
**Language**	English language	Non-English language

### 2.4 Data extraction and charting

Publications identified through the search strategy, were collated on Microsoft Excel and duplicates removed using the remove duplicate function. The study selection process involved the screening of titles and abstracts by two independent reviewers to determine eligibility for full text screening based on the two pre-determined search concepts. English publications were then retrieved for full text review. Publication references were managed using Mendeley Reference Manager.

Consultation with project team leaders who were involved in the Trust implementation team also facilitated and informed the agreement of definitions for overarching publication categorisations to create a useful summary of data, namely Intervention, Evaluation, Perception and Optimisation. Full definitions can be found in Table 2. At the same time, stakeholders also defined the area of focus associated with benefits and challenges of ADC use based on the commonly occurring themes derived from the included papers.

**Table 2:** Definition for overarching categorisation in data summary.

Category	Agreed Definition
**Intervention**	Publications that drew comparisons of pre- and post-implementation of ADC.
**Evaluation**	Publications that studied the impact of ADC on specific, pre-determined dimensions OR Publications which compared outcomes among different forms and areas of ADC.
**Perception of healthcare professional**	Publications that addressed the acceptance and perception of healthcare professionals towards ADC.
**Reference to optimisation**	Publications that proposed optimisation methods to improve ADC practice OR Publications that addressed challenges in ADC practice.

A calibration exercise was then undertaken where 20 % of full-text papers were randomly selected using the *Excel RAND function* for discussion by the two reviewers to determine the content for inclusion in Table 4. An independent review of the overarching categorisation was also undertaken by both reviewers. Any differences were discussed with further refinement to the data set collated to reach a consensus. One reviewer extracted all data from the included papers using a piloted charting table developed to support the extraction and collation of key information from eligible publications. Extracted data fields included: citation (including article characteristics such as year and country of publication), study design, study aim & objectives, setting, medication distribution model, technology infrastructure, key findings, overarching category, and area of focus.

Once data had been extracted from all papers, numerical and thematic analyses was conducted by the lead reviewer. A PRISMA flow diagram was used to report net findings of publication numbers.^6^ Reasons for exclusions were also recorded as part of the full-text review.

## 3 Results

**Fig. 1:**
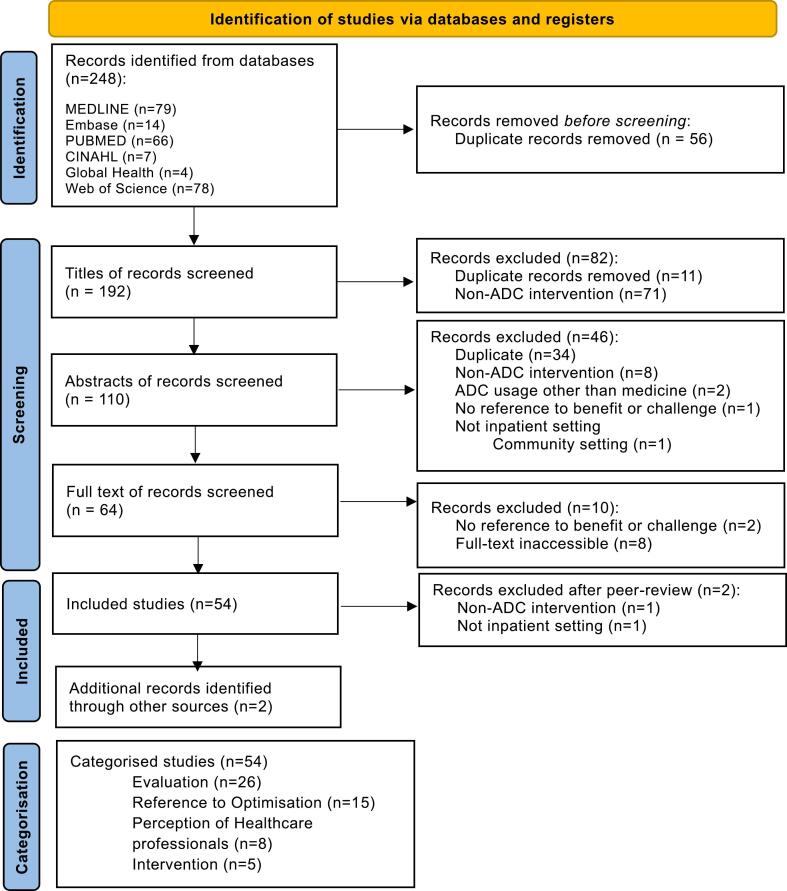
PRISMA flow outlining search and screening process.^7^

Literature search was detailed using the PRISMA flow diagram which outlines the review process and reasons for exclusion in Fig. 1.^7^

### 3.1 Study selection

The literature search yielded a total of 248 publications across six databases. With duplicate publications removed, 192 publications were progressed to the next stages of review. At the title and abstract screening stages, 82 and 46 publications were excluded respectively. Reasons for exclusions can be found in Fig. 1. A total of 64 publications were screened as full text with a further 10 publications excluded. The most common reason for exclusion was due to inability to access the full-text article. An additional two publications were added from external sources. One reviewer extracted all data from the full texts of 54 included publications.

### 3.2 Article characteristics

**Fig. 2:**
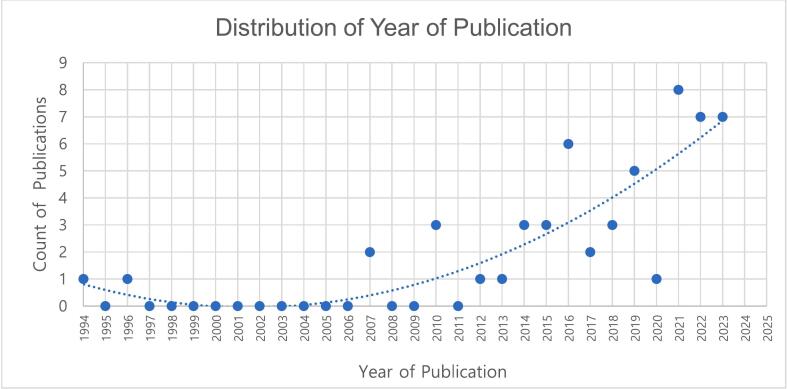
Distribution of included papers according to year of publication.

The review identified 54 papers. The number of papers published on medicines ADC use across hospital inpatient settings has increased significantly in recent years as illustrated in Fig. 2.

Most papers were published in the United States (42.6 %) with a relatively low number of publications originating from the UK (9.3 %); overall 13 countries published research associated with benefits and challenges of ADC use within hospital inpatient settings. The origin of other publications is shown in Table 3.

**Table 3:** Number of publications categorised by country.

Countrry of Publication	Count	Percentage (%)
United States	23	42.6
Australia	6	11.1
United Kingdom	5	9.3
France	4	7.4
Taiwan	3	5.6
Brazil	2	3.7
Canada	2	3.7
Saudi Arabia	2	3.7
Finland	2	3.7
Denmark	2	3.7
Qatar	1	1.9
Colombia	1	1.9
Switzerland	1	1.9
Total	54	100⁎

⁎ The presented percentages sum beyond 100 % due to rounding to 1 decimal point.

### 3.3 Extraction of data

Data extracted from the included publications have been collated in Table 4.

**Table 4:** Extracted data from 54 included studies.

Citation (Author, Country of Publication, Year, Citation number)	Study design	Aim & Objectives	Setting	Distribution Model	Technology infrastructure	Key Findings	Overarching Category	Area of Focus
Ahtiainen et al. Finland (2019)^8^	Systematic review	To review automated and semi-automated drug distribution systems (DDDs) in hospitals to evaluate their effects on medication safety, time and costs of medication care, and to show which of these systems has more advantages compared with others.	Clinical area	Not applicable	Not applicable	Automated drug dispensing systems (ADDS) improved quality of patient care by shifting pharmacists' time from technical distributive activities to clinical work. Automation reduced errors in dispensing and storage, but may produce new safety risks due to changes in processes.	Evaluation	Benefits to cost saving, efficiency saving for nurses and pharmacy technicians, and patient safety
Almalki et al. Saudi Arabia (2023)^9^	Failure mode effect analysis and staff surveys	To aim and describe 1) decrease – the time from ordering medication to administration, including delay incidents, by >70 %; and 2) decrease the inpatient monthly total medication consumption by >20 % and ward medication stock items by >70 %, including decreasing returned items and loss from in-house expired medications by >70 %.	Clinical area	Centralised	ADC	ADC implementation resulted in reduced monthly medicine consumption, ward stock, annual cost, and reported medication delay orders. Workflow efficiency and user satisfaction surveys (nurse and pharmacist) also conveyed good reliability.	Optimisation	Benefits to cost saving, inventory management, patient safety, and efficiency saving for nurses
Alzahrani et al. Saudi Arabia (2023)^10^	Cross-sectional, qualitative study	To identify the issues related to maintaining ADCs in hospitals from the pharmacy technicians' perspective before, during, and after filling and suggest solutions to overcome complications to ADC usability.	Clinical area	Decentralised	ADC	The authors identified the main challenges before, during, and after distribution. These challenges can be minimised by appropriate training of staff.	Perception	Challenges faced by pharmacy technicians in drug distribution (efficiency saving)
Aschenbrenner et al. US (2023)^11^	Article	To emphasise the importance of override safety and alertness.	Clinical area	Not specified	Not described	Frequent use of override function in non-emergency situations compromise patient safety and contribute to medication errors. Users, especially nurses, must engage in safety committees and necessary measures to enhance ADC safety.	Evaluation	Challenges of override function, patient safety
Austin et al. Australia (2018)^12^	Prospective study	To compare medication turnaround times in a paper-based hospital environment with a digital hospital equipped with an integrated closed-loop electronic medication management systems (EMMS).	Clinical area	Centralised	ADC + Computerised physician order entry (CPOE) + Barcode medication administration (BCMA)	Medication turnaround time was less in EMMS site than in paper-based site. This was similar for non-critical medications. This could be owed to increased accessibility of medications through EMMS.	Evaluation	Challenges to efficiency saving and benefits to patient safety
Bagattini et al. Brazil (2022)^13^	Retrospective pre-post study	To describe the outcome of ADC implementation in drug dispensing.	Central pharmacy	Centralised	ADC + Pharmacy dispensing robot	The introduction of ADC reduced frequency of breakage, loss, and expiry of medication and products. Overtime pay for central pharmacy team also reduced. Medication Administration Errors (MAEs) in prescription and distribution phases were observed.	Evaluation	Benefits to efficiency saving and challenges to patient safety
Balka et al. Canada (2007)^14^	Mixed-methods observational study (interview)	To identify challenges to ADC efficiency and suggest essential work context to improve professional practice.	Clinical area	Centralised	ADC + CPOE	Identified issues in workflow hindrance were intravenous (IV) medication refrigeration, inventory control and discrepancy reporting, and implementation in shortage of staff. This failure to consider the social context of ADC technology resulted in partial success of ADC not up to its full potential. The presence of previous nurse practices, such as split dosing, should also be considered.	Optimisation	Challenges to efficiency saving, patient safety, and inventory management
Batson et al. UK (2021)^15^	Systematic review	To identify and summarise published literature regarding clinical and economic value of in-hospital pharmacy automation, including ADC, ADD, and automated dispensing machines (ADM).	Clinical area	Not applicable	Not applicable	Clinical advantages of in-hospital automation included: reduction of MAEs, dispensing time, and refill errors while economic advantages included labour savings, stock and inventory savings, and reduced wastage e.g. expired drugs.	Evaluation	Benefits to efficiency saving by nurses, cost saving, and patient safety
Berdot et al. France (2019)^16^	Quasi-experimental, multicentre	To compare the costs and benefits of an ADC versus traditional floor stock storage (TFSS).	Clinical area	Centralised	ADC + CPOE + Electronic Health Record (EHR)	Nurses' satisfaction with ADCs was high. No medication process error due to storage errors was reported for ADCs, whereas nine were reported for TFSS. On the contrary, informatics-related events increased with the use of ADCs, as expected.	Evaluation	Benefits to cost, efficiency saving by nurses and challenges by informatics-related errors by staff
Burton US (2019)^17^	Mixed methods review	To suggest safe practice recommendations for ADC.	Clinical area (Emergency care)	Not specified	ADC + EHR	An over-reliance on technology and trust in its proper functioning can develop among users. Thus, the risks associated with ADCs must be identified and safeguards in medication storage, return, and selection must be employed interdisciplinarity to promote safe practice instead of contributing to medication errors.	Optimisation	Challenges to override, inventory management, and patient safety
Chapuis et al. France (2010)^18^	Observational, pre-post study	To assess the impact of an automated dispensing system on the incidence of medication errors related to picking, preparation, and administration of drugs in a medical intensive care unit. To evaluate the clinical significance of such errors and user satisfaction.	Clinical area (ICU)	Centralised	ADC	The implementation of ADS reduced overall errors related to picking, preparation, and administration of drugs in Medical Intensive Care Unit (MICU). Most nurses favoured the new drug dispensation organisation.	Evaluation	Benefits to patient safety and user satisfaction
Chapuis et al. France (2015)^19^	Pre-post financial analysis	To evaluate the economic impact of ADS in surgical ICUs.	Clinical area (ICU)	Decentralised	ADC	ADS investment is financially profitable and improves the efficiency of drug. ADS can reduce medication-related costs and nursing time dedicated to medications.	Evaluation	Benefits to cost saving and efficiency saving by nurses
Cottney UK (2014)^20^	Observational pre-post study	To assess whether ADC implementation would reduce MAE and reduce medication administration time in a mental health hospital.	Clinical area (mental health)	Decentralised	ADC	While MAE reductions were not statistically significant, average time taken for nurses to administer a dose of medication decreased from 2.94 min to 2.37 min after the implementation of the ADC, a saving of 0.57 min (*p* = 0.006). At approximately 115 doses administered per ward per day, this would correspond to a total saving of roughly 66 min per ward per day of nursing time.	Intervention	Benefits to efficiency saving in nursing administration time and patient safety
Cousein et al. France (2014)^21^	Observational pre-post study	To assess the efficacy of a daily UDDS and ADC on discrepancies between what is prescribed and what is administered to the patient.	Clinical area (geriatric ward)	Centralised	ADC + Pharmacy dispensing robot	ADC reduced dose MAE by 79.1 % (*p* = 0.005) and drug MAE by 93.7 % (*p* = 0.009) compared to TWWS. In-dept knowledge such as root causes of MAE and cost analysis is necessary to further optimise patient medication management.	Evaluation	Benefits to patient safety by reducing medication error
Craswell et al. Australia (2021)^22^	Qualitative descriptive study (semi-structured interviews)	To explore the structures, processes and outcomes involved in an Automated Medication Dispensing system implementation and its impact on patient safety.	Clinical area	Decentralised	ADC	There is increased stress of implementing multiple new technologies. Hence effective nursing staff training and support are paramount to address inadequate consultation prior to implementation. Further optimisation can be achieved by addressing inadequate hardware and software functionality.	Perception	Benefits to time saving in distribution (efficiency saving) and challenges to user training, inventory management, user unfamiliarity
De-Carvalho et al. Brazil (2017)^23^	Prospective analysis	To assess the economic and patient-safety impact of the implementation of the Pyxis automated materials and drug-dispensing system.	Clinical area (ICU)	Decentralised	ADC	Implementing this technology resulted in reduced human resource costs, improved efficiency of administration staff, reduced audit adjustments regarding drug inclusions, and a lower number of urgent requests and product returns to the central pharmacy.	Evaluation	Benefits to cost saving, patient safety, and efficiency saving by nurses
Epstein et al. US (2016)^24^	Prospective, cohort analysis	To describe a method of capturing drug transactions in near real-time ADC and evaluate subsequent improvement in medication reconciliation accuracy.	Central pharmacy	Decentralised	ADC	Implementation of the near real-time reconciliation reporting process resulted in a decrease in the discrepancy rate between Pyxis and ADC records and anaesthesia Information Management Systems (AIMS) controlled substance transactions compared to the prior epoch of CD.	Optimisation	Challenges in CD governance
Fanning et al. Australia (2016)^25^	Pre-post study	To assess the impact of ADCs on medication selection and preparation error rates in an emergency department (ED).	Clinical area (ED)	Decentralised	ADC	There was a statistically significant reduction in medication selection and preparation error rates pre- and post-intervention (1.96 % and 0.69 % respectively = 0.017).	Evaluation	Benefits to patient safety
Findlay et al. US (2015)^26^	Prospective	To improve ADC inventory management by leveraging dynamic inventory standards and a low inventory alert platform.	Central pharmacy	Decentralised	ADC	Inventory turns were not appreciably changed by dynamic inventory standards, suggesting that stockouts were not reduced simply by overstocking the ADCs. The system's dynamic ability to classify medication dispensing trends as consistent or variable effectively addressed the high number of stockouts without increasing on-hand value of medications. Low inventory alerts may also be useful, while institutional differences must be considered.	Optimisation	Challenges in inventory management
Fox et al. Australia (2021)^27^	Prospective observational study	To investigate the effect of ADC on medication distribution and error rate pre-post automated robots.	Central pharmacy	Decentralised	ADC + Pharmacy dispensing robot	Implementation of ADCs and robots reduced medicine administration time if medicine were stored within the robot. Interoperability among the two systems is vital, otherwise increased time was observed during downtime. Mis-selection of medication reduced post-implementation.	Evaluation	Benefits to efficiency saving for pharmacy staff, patient safety by reduced MAE
Guerrero et al. US (1996)^28^	Prospective analysis	To describe a self-reported work-sampling study of the impact of ADS on medication-related work activities by nurses and pharmacists on two nursing units.	Clinical area (surgical ICU, ED)	Decentralised	ADC	While the proportion of time spent on medication-related activities by nurses did not significantly change, pharmacists gained more time for clinical activities.	Evaluation	Benefits to efficiency saving by pharmacists in medication-related activities
Helmons et al. US (2012)^29^	Prospective study	To assess the effects of a direct refill program for ADCs on medication-refill errors.	Clinical area	Centralised	ADC	A redesign of the ADC refill process (bulk distribution) using a wholesaler-to-ADC direct refill program that included delivery of prepackaged medication and bar-code assisted refill significantly decreased the occurrence of ADC refill errors.	Optimisation	Challenges in inventory management, especially refill errors
Hull et al. US (2010)^30^	Prospective, cohort analysis	To measure data before and after installation of the medication cabinets and compare the number of nurses' steps, trips to Pyxis, nursing frustration, and impact on pharmacy workflow.	Clinical area (progressive care unit)	Decentralised	ADC	Nurse steps per shift did not significantly change pre-post intervention. More inclusion of ADC in assignments demonstrated significantly lower frustration of nurses (*p* = 0.02). Closer observation revealed that frustration arose from missing medications rather than ADC itself.	Perception	Challenges perceived by nurses and pharmacy technician in workflow
Jumeau et al. Switzerland (2021)^31^	Prospective observational study	To evaluate the impact of ADC on dispensing error rate, number of interruptions, and pillbox preparation time.	Clinical area	Decentralised	ADC + CPOE	There was a significantly lower number of error rate (1.0 % vs 5.0 %,*p* = 0.0001) and interruptions per hour (3.2 vs 5.7, *p* = 0.0008) (i.e. colleague speaking, complete interruption) in ADC group than TWSS.	Evaluation	Benefits to patient safety and efficiency saving for nurses
Kelm et al. US (2018)^32^	Single-centre, retrospective-prospective pilot product performance study & prospective satisfaction survey	To describe the findings related to the use of novel ADC.	Clinical area	Decentralised	ADC	New ADC improved user-friendliness, visibility and navigation, storage capacity, and software processing. When frequently co-administered medications were stocked in close proximity to each other, time to administration significantly decreased from 10.5 s to 10.3 s.	Perception	Benefits to efficiency saving for nurses and pharmacists, inventory management
Lichtner et al. UK (2021)^33^	Retrospective, qualitative analysis	To understand the contextual reasons for workarounds or patterns leading to running-out-of-stock at the time of patient need from a study of an ADC in a paediatric intensive care unit (PICU).	Clinical area (PICU)	Decentralised	ADC	Following distribution via Omnicell, PICU nurses reported an ongoing struggle with keeping accurate accounts of medications in the ADC, resulting in running out of stock. It is necessary to understand nurses' information needs, clinical needs of their patients, and context-specific medication work practices, as these contribute to data entry issues and therefore ‘tragedies of the commons’ with the ADC.	Perception	Challenges in inventory management, informatics-related problems by staff
Lichtner et al. UK (2023)^34^	Mixed-methods exploratory study, comprising observations, interviews and audits	To explore whether and how storing CDs in an ADC contributes to the effectiveness and efficiency of CD governance.	Clinical area (ICU)	Decentralised	ADC	Nurses and pharmacists perceived CD governance to be beneficial in safeguarding, documentation, monitoring, and reporting. Challenges also occur in resolving discrepancies, refrigerator management, and drug diversion from discarded medications. CD governance is more complex beyond mere time saving and stringent controls.	Optimisation	Challenges and benefits on CD governance, user satisfaction, governance
Liou et al. Taiwan (2023)^35^	Retrospective study	To assess the effectiveness of ADC on medication distribution process, including delivery time, working time, and transportation labour.	Clinical area (ICU)	Not applicable	ADC + CPOE	There was a significant reduction when converted from centralised to decentralised distribution in drug delivery time due to reduced transport from central pharmacy. Other benefits included reduction in working time of nurses and pharmacists, medication transport labour, reduction of dispensing error incidents, along with satisfaction of users.	Evaluation	Benefits in efficiency saving of nurses and pharmacy staff, nursing satisfaction
Lichtner et al. UK (2021)^36^	Mixed-methods design	To evaluate ward-based processes to control ADC in paediatric ICU (PICU).	Clinical area (PICU)	Decentralised	ADC	Electronic documentation of ADC made CD discrepancies easier to note, improved efficiency of CD governance, and made CD stock count faster than with paper registries.	Evaluation	Benefits to CD governance, efficiency saving of nurse's CD check
Lupi et al. US (2019)^37^	Retrospective analysis	To determine if ADC optimisation by clinical pharmacists would affect the number of dispenses from central pharmacy, number of stockouts, and inventory cost.	Central pharmacy	Not specified	ADC	Benefits can be seen by simple quality improvement efforts by clinical pharmacists. Results saw a reduction in the number of medications that were dispensed from central pharmacy as well as the frequency of medication stockouts on the patient care units.	Evaluation	Benefit to inventory management, efficiency saving, cost saving
McCarthy et al. US (2016)^38^	Prospective	To design an ADC optimisation initiative, including: (1) adjustment of periodic automatic replacement (par) inventory levels (desired on-hand quantities of medications) and par reorder quantities to reduce the risk of ADC supply exhaustion and improve restocking efficiency, (2) expansion of ADC “common stock” (medications assigned to ADC inventories) to increase medication availability at the point of care, and (3) removal of some infrequently prescribed medications from ADCs to reduce the likelihood of product expiration	Clinical area	Not specified	ADC	Results observed were reduced pharmacy technician labour, decreased stockout percentages, generated opportunities for cost avoidance, and improved medication turnaround times. While ADC can confer such benefits, it is essential that available data from implementation is reviewed regularly and user re-education is repeated to overcome challenges.	Optimisation	Benefits to inventory management and cost saving via optimisation
Metsämuuronen et al. Finland (2020)^39^	Observational, survey	To investigate nurses' perceptions of ADCs and the impacts of ADCs on nurses' work.	Clinical area (surgical unit, ICU)	Not specified	ADC	Nurse's use of time was more efficient post-ADC, as demonstrated by reduced dispensing and preparation time by average 32 min per 8 h shift. Features of ADCs that improve patient safety were barcode scanning, greater confidence in medicine selection, light guidance, and the informative medicine labels generated by ADC.	Perception	Benefits to nurses efficiency saving by reduced dispensing time, patient safety, and user satisfaction and challenges by resistance to change
O'Neil et al. US (2016)^40^	Prospective	To compare two methods of optimising ADCs, which are adjusting par levels according to average daily usage and adjusting par levels according to standard inventory formula.	Clinical area	Decentralised	ADC	Three steps of optimisation of inventory management via: (1) removal of medications that had not been dispensed for at least 180 days, (2) movement of ADC stock to better suit end-user needs and available space, and (3) adjustment of par levels (desired on-hand inventory levels) by daily usage or standard formula were tested. Optimisation of ADCs using a standard inventory formula had a significant impact on vend: fill ratios (*p* = 0.0477), while other comparisons were statistically insignificant.	Optimisation	Challenges of inventory optimisation and cost saving
Oldland et al. US (2015)^41^	Prospective study	To measure the effects associated with sequential implementation of electronic medication storage and inventory systems and product verification devices on pharmacy technical accuracy and rates of potential medication dispensing errors	Central pharmacy	Centralised	ADC + BCMA	Clear benefit in ME was only observed after intense user training was provided to all technical staff regarding correct application and consistent use. Procedural changes involving barcode technology and re-training strategy reduced quantity errors by more than 50 %.	Evaluation	Challenges to user training and patient safety
Paterson et al. US (2022)^42^	Retrospective, observational	To discover whether overridden medications were being administered before verification (indicating it was needed emergently, thus justifying override) or after verification (indicating the override did not result in quicker administration and/or the medication was not emergent).	Clinical area (ED)	Decentralised	ADC + EHR	Approximately 27 % of the overridden medications were overridden unnecessarily because these prescriptions were actually verified quickly enough to be administered at or before their actual time of administration without override. Regular re-education of override protocols and risks should be conducted to ADC users to ensure that a ‘habit of overriding medicines’ does not develop, and a verbal/written prescription should be entered before override.	Evaluation	Challenges to override use and governance
Repella et al. US (2022)^43^	Retrospective	To reduce system overrides, educate multi-disciplinary departments regarding the use of appropriate overrides, and create a method to review ADC overrides.	Clinical area (ED)	Decentralised	ADC + CPOE	Override functionality in ADC can be optimised via regular review of overridable medicines to accommodate practice, continual data review, interdisciplinary communication based on reviewed data to improve practice, and expand education for nurses.	Optimisation	Challenges of override use, governance, user training
Rhodes et al. US (2019)^44^	Article	To identify issues related to ADC override and propose methods to improve override functionality.	Clinical area	Not applicable	ADC	Override access based on user-selected indication was suggested as a way of reducing medication errors. This functionality could prevent look and sound alike medication errors, verbal request errors, and consider human factor errors to enhance patient safety.	Optimisation	Challenges to override governance and patient safety
Rhodes et al. US (2022)^45^	Quasi-experimental, retrospective study	To evaluate the impact of ADC functionality expansion on technology-induced errors such as controlled substance discrepancies.	Clinical area	Not specified	ADC	While cassette expansion led to a significant increase in cassette transactions and led to significantly reduced CD discrepancies, there was an increase in dispensing errors post-expansion.	Optimisation	Challenges to controlled drug (CD) governance and patient safety
Rhodes et al. US (2022)^46^	Prospective, qualitative analysis	To describe a pharmacist-led reconciliation process for automated dispensing cabinet (ADC) medication override setting maintenance.	Clinical area	Not specified	ADC	Policy updates resulted in an increase in the number of medications approved for override dispenses from 80 to 106 total medications. The total number of ADC dispense settings requiring maintenance and configuration by pharmacy automation specialists was reduced from 5600 to 541.	Optimisation	Benefits to patient safety, governance, override function
Risør et al. Denmark (2017)^47^	Prospective, controlled cohort study	To evaluate the cost-effectiveness of an automated medication system (AMS) implemented in a Danish hospital setting with regards to electronic medication administration record (eMAR), automated medication dispensing system (AMS), and bar code medication administration (BCMA).	Clinical area (haematological wards)	Centralised	ADC + eMAR + BCMA	Closed-loop medication administration reduced the proportion of medication administration errors. The cost-effectiveness analysis showed that while there was increased AMS implementation costs of €16,843 per 6 months, reduced administration errors made this a worthy investment.	Evaluation	Benefits in patient safety and cost saving
Risør et al. Denmark (2018)^48^	Controlled analysis	To evaluate the effectiveness of two automated medication systems ((i) a complex automated medication system (cAMS) consisting of an automated dispensing cabinet, automated unit-dose dispensing and barcode medication administration (BCMA) and (ii) a non-patient-specific automated medication system (npsAMS) consisting of automated UDDS and BCMA) in reducing the medication administration error rate in comparison with current practice	Clinical area (acute wards)	Centralised	ADC + BCMA + eMAR	cAMS effectively reduced the portion of administration errors driven by procedural errors. npsAMS showed a significant decrease of clinical error compared to the control ward.	Evaluation	Benefits in patient safety
Roman et al. Australia (2016)^49^	Time and motion method	To examine the change in medication retrieval times, number of medications retrieved and staff perceptions before and after the installation of automated dispensing machines.	Clinical area (ED and trauma centre)	Decentralised	ADC	ADC improved more restricted CD management efficiency and reduced retrieval time by replacing manual documentation, which is clinically significant in emergency departments. However, unscheduled medications required longer retrieval time with ADM due to login in and entering patient detail. Despite this, users perceived ADC to help with efficiency.	Evaluation	Benefits to CD administration time (efficiency saving), user perception and challenges to non-scheduled medicine administration time (efficiency saving)
Shermock et al. US (2023)^50^	Comparative analysis	To describe and compare U.S and Finnish hospitals' EMMS approaches and their impact on medication safety and workflows.	Clinical area	Both	ADC + BCMA + eMAR	From comparison of centralised US hospitals and decentralised Finnish hospitals, it was understood that interoperability between EMMS systems is crucial. ADC implementation should be continuously developed in consideration of institutional differences and continuous performance improvement.	Evaluation	Benefits to patient safety, inventory management, governance
Sirois et al. Canada (2013)^51^	Semi-structured interviews	To explore nurses' perceptions and attitudes towards current technology use on their units and towards the introduction of automated dispensing units (ADUs) technology and use with nursing staff in two different hospitals.	Clinical area	Decentralised	ADC	All participants stressed the importance of pre-implementation training. ADC has been identified as impractical in an emergency room setting or to monitor an unstable patient's condition.	Perception	Benefits to user satisfaction (staff efficiency)
Skibinski et al. US (2007)^52^	Mixed-methods pre-post study (questionnaire, interview)	To assess the effects and outcomes of implementing new technology into the medication-use process.	Clinical area (ICU)	Decentralised	ADC + CPOE	Despite perceptions that technology complicated medication access, medication administration became more accurate and efficient, eMAR accuracy improved, and dispensing processes became facilitated.	Intervention	Benefits to patient safety, efficiency saving for nurses, pharmacy technicians, and other ADC users
Taylor et al. US (2022)^53^	Article	To describe a fatality related to ADC and suggest methods to prevent such errors.	Clinical area	Not specified	Not described	Two major suggestions were provided: removing neuromuscular blocking medications from ADC (limiting access of medicines to appropriately trained personnel) and optimising ADC functionality such as limiting use of trade names, utilising five-letter search, and scrutinization of override usage. There is no one-fits-all approach, and constructive re-engineering of ADC usage is necessary.	Optimisation	Challenges to patient safety, override function (governance)
Tsao et al. Columbia (2014)^54^	Systematic review	To summarise and evaluate the existing literature reporting The clinical and economic impacts of using decentralised adds in hospitals.	Clinical area	Decentralised	Not applicable	The efficacy of ADC on nurse's time, pharmacy technician time, pharmacist time, and cost largely depends on institutions' medicine administration and dispensing process, stressing the importance of proper integration.	Evaluation	Benefits to efficiency saving for nurses and pharmacy technicians, cost saving
Tu et al. Taiwan (2023)^55^	Prospective cohort analysis	To assess the benefits of ADCs by comparing the rates of medication errors, including prescription, dispensing, and administrative, before and after their adoption.	Clinical area (ICU)	Decentralised	ADC + CPOE + EHR	The adoption of ADCs effectively reduced medication errors in the ICU: the dispensing error rate reduced from 3.87 to 0 per 100,000 dispensations (*p* = 0.003). ADCs were also effective in reducing errors that caused no harm as listed in National Coordinating Council for Medication Error Reporting and Prevention (NCC MERP) categories B—D.	Intervention	Benefits to patient safety, efficiency saving for nurse and pharmacist, override function (governance)
Wai et al. US (2022)^56^	Pre-post study	To determine the impact of a business intelligence dashboard tool to optimise ADCs	Clinical area	Decentralised	ADC + Pharmacy dispensing robot	(1) removal of unused medications over 90 days (2) adjusting periodic automatic replenishment (PAR) Levels (3) addition of commonly dispensed medications Resulted in reduced inventory cost, percentage of stockout, and average of missing doses.	Optimisation	Benefits to inventory management and cost saving
Wakefield et al. US (2010)^57^	Interview	To describe the experience of rural hospitals where the duty of medication filling, retrieval, and administration duties were transferred to patient care nurses due to shortage of pharmacists.	Clinical area (critical access hospitals)	Decentralised	ADC + CPOE + EHR	The importance of collaboration among healthcare professionals was noted. These hospitals have improved medication process Quality and safety by changing how medications are retrieved and by providing automated point-of-care medication administration checking and automated billing.	Evaluation	Benefits to patient safety
Wang et al. Taiwan (2021)^58^	Longitudinal, observational	To determine the impact of an ADC system on medication administration by nurses as well as safety before and after ADC implementation.	Clinical area	Decentralised	ADC + eMAR + CPOE	The majority of nurses were satisfied with the system, but there was a negative impact on workflow relating to access to medications. Product labelling and more staff training in the use of barcode systems were associated with a decrease in the rate of medication error to 0.050 %.	Intervention	Benefits to patient safety and efficiency saving for nurses
Williams et al. US (1994)^59^	Retrospective study	To describe the impact of AMS with labour productivity and reduction in lost charges as key indicators.	Clinical area	Not specified	Not described	The introduction of AMS induced 2.38 workload hour reduction per patient day and estimated saving of $192,448 annually, exceeding expectations. Nurse satisfaction and perception of increased security with the system was observed.	Intervention	Benefits to efficiency saving for nurses, cost saving, user satisfaction
Zaiden et al. Qatar (2016)^60^	Piloted, validated, online, and anonymous questionnaire	To assess nurses' perceptions of and satisfaction with the use of ADCs.	Clinical area (Heart Hospital Centre for Cancer Care and Research)	Decentralised	ADC	Overall, nursing staff were satisfied with the use of the technology and believed it facilitated their work and could contribute to safer healthcare and possible reduction in medication errors and “near misses”. The most frequent suggestions made were to make all formulary medication available in the ADC system (*n* = 60), for the pharmacy to enter prescriptions quickly (*n* = 55), to install more machines in certain units (*n* = 37), and to replace the open matrix drawers with locked lidded drawers (*n* = 16).	Perception	Benefits to user satisfaction, efficiency saving for nurses
Zheng et al. Australia (2021)^61^	Systematic review	To determine the impact of ADCs, BCMA and closed-loop EMMS on clinical work processes, medication safety, and drug diversion associated with controlled medications in the inpatient setting.	Clinical area	Decentralised	Not applicable	ADCs reduced MAEs and preventable adverse drug events related to controlled medications. Results showed that ADCs can support monitoring of controlled medications by eliminating load and unload error during stocking and removing of medications. ADC alone is not sufficient to prevent drug diversion.	Evaluation	Benefits to CD governance, patient safety, challenges to governance

### 3.4 Description of setting, medicines distribution model, technology infrastructure and overarching category

Most included papers (*n* = 48, 88.9 %) addressed ADC use in clinical ward areas in inpatient hospital settings. Only six (11.1 %) studies focused on ADCs located in dispensaries in central pharmacy.

Different ADC medicine distribution models, and associated workflows, were described across the literature. A centralised model often refers to medications being located centrally in one ADC location, typically within the pharmacy department, with medicines distributed and dispensed for individual patients. Whereas a decentralised model refers to medications' distribution from central pharmacy to multiple ADCs located in clinical areas enabling medicines to be stored and tracked securely enabling nurses to accurately obtain patient-specific medication on demand.^62^ In the reviewed literature, this was defined as either centralised (*n* = 11, 20.4 %) or decentralised (*n* = 29, 53.7 %) models of distribution.

When considering technology infrastructure and solutions described in literature, ADC alone accounted for 50.0 % (*n* = 27); ADC with computerised physician order entry (CPOE) 9.3 % (*n* = 5); ADC with pharmacy dispensing robot 7.4 % (*n* = 4); ADC with electronic health record (EHR) 3.7 % (n = 2), and ADC with barcode medication administration (BCMA) 1.9 % (*n* = 1). Publications which described multiple integration of three or more technology solutions accounted for 14.8 % (*n* = 8) of studies reviewed. For a proportion of papers, the infrastructure was not described (*n* = 3, 5.6 %) or not applicable (*n* = 4, 7.4 %), where the nature of the papers were systematic reviews with varying technology infrastructure therefore deemed inapplicable.

Twenty-six of the included publications were categorised as evaluation papers (48.1 %), 15 optimisation papers (27.8 %), eight perception papers (14.8 %), and five intervention papers (9.3 %). It was notable that all eight perception publications focused on the use of ADCs with reference to staff efficiencies.

The selected papers were further subcategorised into themes as outlined in Table 5. Thirty-two (59.3 %) publications explicitly discussed benefits whilst fifteen publications (27.8 %) addressed challenges with ADC use for medicines. Seven papers (13.0 %) looked at both benefits and challenges. Of note, many publications had more than one area of focus.

**Table 5:** Area of focus distribution per publications which addressed benefits, challenges, or both.

	**Area of Focus**				
	Patient safety	Inventory management	Staff efficiencies	Cost saving	Governance
Benefits	18	6	19	11	4
Challenges	7	6	3	1	6
Both	2	1	6	0	2

When considering the benefits papers, the main areas of focus were staff efficiencies (*n* = 19, 32.8 %), patient safety (*n* = 18, 31.0 %), and cost savings (n = 11, 19.0 %). The main areas of focus described in challenge papers were patient safety (*n* = 7, 30.4 %), inventory management (*n* = 6, 26.1 %), and governance (*n* = 6, 26.1 %). In publications that described benefits and challenges, staff efficiencies were the most prevalent topic of focus (n = 6, 54.5 %).

### 3.5 Benefits

#### 3.5.1 Staff efficiencies

Among the 25 publications which addressed the benefits of ADCs relating to staff efficiencies: 14 publications described the benefits associated with nursing efficiency savings (56.0 %), six referenced both nursing and pharmacy staff efficiency savings (24.0 %), and five publications focused on pharmacy staff efficiency savings alone (20.0 %). Chapuis et al. described that nursing time dedicated to selecting, preparing, and administering medication reduced post-ADC implementation with a mean time gain of 14.7 h per day across three intensive care units (ICUs) (4 h/9 beds).^19^ This observation was supported by other publications,^15^^,^^19^^,^^20^^,^^23^^,^^35^^,^^39^ where Cottney et al. described 66 min of time saving associated with medicines administration per ward per day by nurses (2.94 min pre-ADC to 2.37 min post-ADC).^20^ De-Carvalho et al. reported a reduction in nursing time spent on stock inventory counting with 6 h saved daily post-ADC implementation.^23^ Metsämuuronen et al. reported a 32 min reduction of nursing time per 8-h shift in the retrieval and preparation of medications.^39^ It was also reported that nurses experienced less interruptions such as colleague speaking reduced from 5.7 pre-ADC to 3.2 post-ADC (*p* = 0.0008) per hour.^31^

CD discrepancies were also reported to be easily identified by nurses.^36^ The time taken for nurses to undertake CD counts was perceived to have reduced significantly, however, this was not quantified.^34^ Roman et al. reported nursing efficiency savings resulting from a reduction in the time taken to retrieve CDs from ADCs whereby the requirement to document the transaction was removed as it was automatically captured in the ADC electronic CD register.^49^

Pharmacy efficiency savings were described in terms of pharmacist and pharmacy technician time attributed to medicines management and distribution activities. As a result of ADC implementation pharmacy staff reported more time available for clinical activities, leading to an improvement in the overall quality of care delivered to patients.^8^^,^^23^^,^^27^^,^^28^^,^^32^^,^^52^^,^^54^ Liou et al. noted that pharmacists spent on average 5.6 ± 0.2 h less per month dealing with requests for medication supply.^35^

#### 3.5.2 Patient safety

Improvements to patient safety were described in literature in a variety of different ways, including the reduction of medication administration errors (MAEs) as well as a reduction in the inaccuracy in stock inventory rates, which in turn had the potential to lead to omitted doses. It was described that ADC implementation resulted in a 79.1 % (*p* = 0.005) reduction of wrong dose and drug MAEs specifically by 93.7 % (*p* = 0.009),^21^ but it was also noted that knowledge on the root causes of MAE is crucial to further improve medication management to enhance patient safety benefit.^21^^,^^55^ Jumeau et al. further reported that ADC implementation reduced number of error rates from 5.0 % to 1.0 % (*p* = 0.0001).^31^ Medicine discrepancy rate refers to inconsistencies among documented lists of medicines regarding dosage, stock, formulation, and frequency. Further reduction in MAE rate was observed^15^ to 0.05 % following ADC implementation and appropriate product labelling with barcode systems.^58^ It was highlighted that while there were reduced errors, changes in procedures and systems may incur a new set of risks.^8^^,^^15^

#### 3.5.3 Cost saving

Benefits in cost saving were observed from reduced ward stock supply and annual spend on expired medicines, decreased stockouts, and decreased stock orders from central pharmacy [8,9,19,37,38,56,63]. McCarthy et al. recommended that regular data review along with user re-education is essential to effectively utilise the information gathered from technology implementation.^38^ Tsao et al. observed that mean charge capture rates of medicines improved from 63 % pre-ADC to 97 % post-ADC, leading healthcare providers to bill for a larger proportion of medications used, thus improving revenue accrual rates.^54^ McCarthy et al. estimated cost avoidances to $19,660 from circumventing 835 infrequently used items to central pharmacy and avoiding expiration.^38^

Benefits in cost savings were also described as a reduction in staffing costs [13,23,38,54,59,63]. Bagattini et al. observed direct cost saving from reduced overtime payment for central pharmacy team.^13^ De-Carvalho et al. interpreted cost savings from reduced staff time spent on various activities, which corresponded to reduced staffing costs. Savings were calculated according to mean gross salary and reported as follows: full-time nurse (R$ 5869; USD 2523), administrative/hospitality assistant (R$ 1598; USD 687), materials supervisor (R$ 3526; USD 1516), and pharmacy assistant (R$ 1710; USD 735) per year during the first year after introducing the ADC.^23^ This was explained as a redistribution of time, where time spent by nurses and administrative assistants on medication related activities decreased; however, it was noted that the time spent on stock refill and inventory activities performed by pharmacy assistants increased.^23^ McCarthy et al. observed reduced pharmacy technician labour requirements.^38^ Williams et al. observed a reduction in nursing and pharmacy workload of 2.38 h per patient per day and estimated savings in the range of $192,448 annually, including a significant reduction in costs attributed to lost inventory ($542.98 monthly pre-ADC to $1.82 3-months post-ADC).^59^

Risør et al. conducted a cost-effectiveness analysis over a six month period following ADC implementation. Costs savings associated with medication error prevention were reviewed by comparing number of errors in the medication administration process before and after implementation.^47^ A reduction of medication administration error ratio pre and post implementation from 0.35 to 0.17 was reported. Calculations of ADC implementation cost per 100 patient days (€1117 per 100 patient-days) reported to be higher than that of conventional medication management (€615 per 100 patient-days), corresponding to incremental costs of €502 per 100 patient-days.^47^ However, Risør et al. added that despite increased costs, potential cost savings of avoided MAEs of 258 avoided administration errors, 178 procedural errors, and 27 clinical errors per 100 patient-days could balance the incremental costs.^47^

### 3.6 Challenges associated with ADC use in medicines

#### 3.6.1 Patient safety

Bagattini et al. described the benefits of streamlining medication selection with ADCs in reducing MAEs however also highlighted that prescribing and distribution errors persisted.^13^ Similarly, Balka et al. identified that reluctance to report discrepancies and staff shortages during ADC implementation could lead to ineffective use of ADCs, which potentially impacts patient safety due to unresolved or unreported discrepancies and improper use of the system.^15^ Aschenbrenner et al. discussed how frequent non-urgent use of the override function compromises patient safety, as it bypasses pharmacist verification and safety measures intrinsic to the system, and describes a well-known adverse event, where vecuronium, a smooth muscle relaxant, was erroneously selected via the manual override function instead of the intended drug Versed (brand name for midazolam, a benzodiazepine medication).^11^ Taylor et al. suggested that ADC functionality should be optimised by limiting the use of trade names, using 5-letter search method, and scrutinising override use to strengthen patient safety and reduce risk of errors.^53^ Oldland et al. reinforced the importance of user training by stating that MAEs were reduced only, once intense user education on accurate use of ADCs was undertaken.^41^ Balka et al. noted that when IV stock medication intended for refrigeration was replenished, this was insufficiently communicated to nursing staff. This led to these fridge items being left in non-refrigerated areas which rendered them unusable and caused potential missed doses and resulting patient safety implications.^14^

Burton et al. observed overreliance on ADC technology among users in the Emergency Department (ED), and preventative actions were suggested in order to enhance patient safety surrounding high risk medications.^17^ Measures included stocklist rationalisation whereby high-risk medications e.g. methotrexate and fentanyl were removed. These medications would need to be requested from central pharmacy thereby necessitating a pharmacist to screen the order prior to supply.^17^

#### 3.6.2 Inventory management

Challenges associated with inventory management of medicines which require special storage requirements have been described in published literature. Balka et al. identified IV medication stock management as a challenge, as IV medications were occasionally stocked in alternate locations such as ADC cabinets instead of in ADC-controlled refrigerators.^14^ Lichtner et al. identified that challenges also occur in resolving CD discrepancies whereby refrigerator CD lines can be removed by any registered user accessing other medications in the fridge without a witness.^34^ De-Carvalho et al. described that item refilling and stock inventory management was newly allocated to pharmacy assistants, increasing time spent from 0 h per day to 1 h/day and 3 h/day respectively.^23^

Medication management was also complicated by user-related challenges. Lichtner et al. highlighted nurses often faced stockouts and discrepancies with stock medicines, which were thought to be multifactorial including difficulties in correcting stock levels due to unfamiliarity with the system.^33^ Hull et al. also noted that nurse dissatisfaction surrounding ADCs arose from medication stockouts.^30^ Moreover, Alzahrani et al. identified challenges faced by pharmacy technicians before, during, and after introduction of a decentralised ADC medication distribution.^10^ Stockouts was the main issue pre-ADC, discrepancies on stock levels during implementation, and unnecessary stock orders from central pharmacy once ADCs were implemented.

A prevalent method of addressing such user challenges was robust staff training.^14^^,^^22^^,^^33^ While re-education was emphasised in studies, Lichtner et al. stressed that nurses' information needs, patients' clinical needs, context-specific medication work processes, and common work practices largely contribute to ADC challenges, hence needs to be considered.^33^ Burton et al. emphasised the importance of safeguards in medication storage, return, and selection to promote safe and effective ADC practice, which should be a coordinated, multidisciplinary effort.^17^ Craswell et al. pointed out that challenges regarding new technology is foreseeable, and that continuous optimisation is required to address such challenges.^22^ Further, Lichtner et al. identified that there was a blame culture directed towards nurses, where pharmacy staff considered stockouts to be caused by “nurses' unwillingness to comply with data entry rules”.^33^

#### 3.6.3 Override function

The unnecessarily frequent use of override function was described as a challenge and explored in many studies.^11^^,^^42^^,^^43^^,^^45^ Aschenbrenner et al. described how frequent non-urgent use of the override functionality compromises patient safety by bypassing pharmacist verification in the medication use process.^11^ Repella et al. supports this notion by stressing the importance of reducing non-emergency override transactions to reduce the risk of a potential adverse drug event.^43^ Repella et al. also shared insight into possible causes for increased override rates, such as misalignment between Standard Operating Procedures (SOP) and confusion regarding permissible overrides; both factors which may be caused by insufficient training.^43^^,^^45^ Craswell et al. further noted that inconveniences in ADC usage, such as queuing, could lead to increased workaround practices by nurses – medication overrides being the main example discussed.^22^

It was mentioned that a means of addressing ‘habitual overriding’ was to ensure that verbal or written prescriptions are prescribed in a timely manner to prevent override transactions.^42^ Rhodes et al. (2019) suggested indication-based override access as a way of potentially preventing MAEs. This application could help to identity the reasons why nurses are utilising the override function to allow the system administrator to troubleshoot any technical fixes needed in order to reduce the frequency of override transactions thus promoting patient safety.^44^

#### 3.6.4 Controlled drugs

Of the included publications, five addressed CD governance (*n* = 6,11.1 %). Lichtner et al. reported challenges in resolving CD discrepancies, refrigerator management, and drug diversion from discarded medications.^34^ Zheng et al. noted that while ADCs facilitate CD governance, ADC alone is not sufficient to prevent drug diversion.^61^ Rhodes et al. observed the impact on technology-induced CD discrepancies by expanding ADC functionality.^45^ This involved direct inventory reassignment of medications from locked bins to ADCs, which resulted in expanded CD cassette transactions and significantly reduced stock discrepancies at the expense of increased dispensing errors.^45^

### 3.7 Other considerations

To maximise the appropriate use of ADCs and adherence to the intended workflows, the provision of effective nurse training and support is important. This includes ensuring that the timeliness of training to correspond with implementation to maximise education efficacy.^22^ Interoperability between ADCs and other electronic systems in use in hospitals is also crucial to maximise systems functionality and capability.^50^ Furthermore, the importance of a continuous cycle of improvement for both hardware and software functionality was noted to be of importance but not explored in detail.^22^

Benefits regarding CD governance was observed as well, where nurses and pharmacists perceived ADCs to be beneficial in CD safeguarding, documentation, monitoring, and reporting.^34^ Further, Zheng et al. supported that ADCs support CD governance by facilitating procedures such as monitoring of controlled medications and reducing errors during stocking and removing of medications.^61^ Epstein et al. reported that simulated near-real time transaction reconciliation processes of comparing ADC and medication administration records also successfully reduced CD discrepancy rates.^24^

## 4 Discussion

Our findings suggest that the type of ADC medicines distribution model and associated workflows deployed unsurprisingly varies between countries. This is possibly reflective of the respective healthcare systems including how they are funded. As an example, there may be a need to operate a centralised medicines distribution model in the United States where each medicine dose unit supplied to patients is charged and private insurance is commonly relied upon to fund healthcare. This is in comparison to the decentralised medicines distribution model observed in UK hospitals, where healthcare provided by the National Health Service (NHS) is generally free at the point of use; a healthcare system funded through taxation. The heterogeneity of medicine distribution models between countries means that not all published benefits and or challenges are applicable to all settings. More research with clear detail regarding study setting and study design would help to inform practice in the UK where the use of ADCs is becoming more commonplace.

This scoping review has categorised ADC publications according to study types: intervention, evaluation, perception, and optimisation and further specified the key areas of focus of each publication. The breadth of literature reviewed and the way it has been consolidated allows hospitals considering ADC implementation to foresee and better plan for challenges and to understand, more fully, the benefits ADCs bring. The differences between distribution models, workflows and technology infrastructure described does, however, limit the generalisability of findings. Further research with pre-and post-ADC intervention design may be of value in helping to inform hospitals planning on implementing ADCs of the likely benefits and potential barriers that could inform practice in similar settings. Studies should include a clear description of digital technology used in organisations to enable transferability of results.

For any hospital considering the implementation of ADCs, recognising the associated benefits and challenges is important to support achieving optimal outcomes. While it was emphasised in many studies that ADCs improve staff efficiency by automating manual procedures, it is essential that organisations understand that new activities and associated tasks also arise from the use of ADCs. This is a factor that needs to be taken into consideration by organisations who should exercise caution if deliberating headcount cuts as a way of offsetting implementation costs. De-Carvalho et al. recommended how time and productivity savings should be redistributed to patient-centred interactions.^29^ The types of tasks which have been shown to take more time post ADC implementation included item spent refilling and reorganising stock inventory; both of which are undertaken by pharmacy staff,^23^ and retrieval of items causing nursing queues at the cabinet.^22^ The relatively small numbers of publications that looked at long-term use of ADCs is also worth highlighting at this point. An alternative angle to consider would be return on investment from stock inventory management over the lifecycle of the hardware; with patient safety and potential redistribution of staffing time to clinical care being other added benefits. Specific UK studies considering return on investment in the longer run would be of value going forward to make the case for ADCs more robust. Further, cost avoidance could be studied in further detail for a set time frame.

The introduction of any new technology can introduce new risks. Patient safety could be improved by addressing safety concerns identified from the literature, namely override usage, governance gaps and poor compliance to new workflows that ultimately impacts inventory management and the timely availability of medicines for patients. All these concerns can be addressed through effective models of training, continuous cycles of optimisation and improved monitoring through report utilisation.^10^^,^^34^^,^^43^ Safety concerns associated with the unnecessary use of override functions was a key concern. In practice, override functions are necessary to prevent delays in medicine retrieval. Being an integral functionality of ADCs, local procedures should clearly define the instances where use of overrides are accepted. The ability to regularly review and report on override transactions also supports providing assurance on compliance with workflows whilst helping to identify any potential issues with the system so that pharmacy staff can operate in more of a prospective rather than retrospective manner. These reports should be shared with nursing colleagues, as needed, to address anomalies in day-to-day practice and identify areas that may benefit from further targeted training.

Only a small number of ADC publications focused on the management of CDs. Publications not only described the benefits of ADC use with CDs e.g. automated recording of CD transactions by user and associated nursing time savings but also focussed on the governance challenges experienced. The settings of these publications were mainly in clinical areas. Further research on the use of ADCs for the management of CDs in the pharmacy setting or across pharmacy and clinical areas would be helpful.

Further advances in ADC functionality and interoperability can also be fostered through continued, collaborative working with system suppliers. This includes evolving technology improvements and enhancements, workflows, as well as proactive monitoring that will support organisations keep the system safe for patients, efficient for providers and complaint with legal requirements. It is further important to note that providers are key stake holders in maintenance and optimisation of ADC in future. Continuous cycles of purposeful reporting, auditing, and data analysis are essential to maximise the benefits of ADCs. Repella et al. observed that reviewing transactions was helpful in ensuring that procedures were being adhered to.^43^ It should also be recognised that ADC optimisation is a continuous process which requires engagement by all users of the system as well as leadership from senior nursing and pharmacy teams. Further research investigating optimum stock optimisation methods and appropriate intervals at which these should be undertaken would help to further improve medicines inventory management. This scoping paper demonstrates that there has been an increase in the number of other technologies that ADCs exist with since their establishment over 30 years ago. Better interoperability of ADCs with other technologies may require further software developments to be undertaken by the providers. This cannot be done in isolation and must be a collaborative effort between ADC providers and the systems they are integrating with.

A good training plan including post-implementation support, regular review of utilisation and adherence to policies and procedures, refresher and best practice updates are important when introducing new technologies. Although there is literature on the benefits of continuous cycles of training with ADC implementation,^33^ no studies have recommended the frequency with which training should be undertaken. Furthermore, the details of training delivery methods and training content have not been sufficiently explored. These are all factors that should be considered by hospitals planning to introduce ADCs; shared learning from hospitals that have implemented and/or supplier standardised training support could help bridge this gap making implementation and system maintenance more streamlined.

### 4.1 Strengths and limitations

The primary strength of this scoping review is its comprehensive nature and breadth. We utilised scoping review methodology according to Arksey and O'Malley^5^ to examine and report on the extent and range of benefits and challenges associated with ADC use for medicines. We comprehensively assessed trends in dates, geographic locations and healthcare settings of research and also reviewed study types; summarising associated outcomes published.

Although this study was conducted in accordance with scoping review methodology, there are some limitations worth noting. Firstly, we aimed to provide an entire scope of all research on benefits and challenges of ADC use for medicines in hospitals to enable the synthesis of as broad a scope of this literature as possible; we did not conduct a formal quality assessment of included studies. We were therefore unable to provide further analysis of general trends beyond the categorisations applied. Secondly, full-text extraction of data was only undertaken by one reviewer owing to the number of studies identified. That said, two independent reviewers screened title and abstracts to determine eligibility for inclusion in the review and a standardised data charting table was used to summarise key findings to ensure consistency of information collated. In addition, 20 % of papers were randomly selected for discussion between the two reviewers to determine the content for inclusion to support validation of the piece. Lastly, a key limitation of the current scoping review is a challenge common to review studies where it is important to acknowledge that in any growing field of research, the results of this scoping review only represent a snapshot at a particular point in time.

## 5 Conclusion

This scoping review was able to examine, categorise and describe ADC literature providing an overview of published benefits and challenges, making it a useful summary for hospitals planning to introduce these systems. Through this review, we were able to identify key gaps in literature specifically focusing on benefits and challenges of ADC use with medicines, including the need for further research in the UK. As more NHS hospitals implement ADC technology the definition of outcomes achieved is useful to further substantiate findings from studies in other countries. More research on longer term benefits and challenges is imperative to not only track the impact of ADCs over time but also to inform practice on keeping technology optimised to continue delivering anticipated benefits.

## CRediT authorship contribution statement

**Yoo Young Jung:** Writing – review & editing, Writing – original draft, Visualization, Validation, Supervision, Software, Resources, Project administration, Methodology, Investigation, Formal analysis, Data curation, Conceptualization. **Áine Walsh:** Writing – review & editing, Visualization, Validation, Supervision, Software, Resources, Project administration, Methodology, Formal analysis, Conceptualization. **Jig Patel:** Writing – review & editing, Supervision, Methodology. **Kit Lai:** Writing – review & editing, Visualization, Validation, Supervision, Resources, Project administration, Methodology, Investigation, Formal analysis, Conceptualization.

## Declaration of competing interest

The authors declare that they have no known competing financial interests or personal relationships that could have appeared to influence the work reported in this paper.

## Appendix A Supplementary data

Supplementary material Image 1
