# Supplementary material for: Benefits and challenges associated with implementation and ongoing use of automated dispensing cabinet for medicines: A scoping review
Source: Explor Res Clin Soc Pharm. 2025 Apr 1;18:100599. doi: 10.1016/j.rcsop.2025.100599 (PMC12005321; doi:10.1016/j.rcsop.2025.100599)
Supplement: Supplementary file 1 — Supplementary material [file mmc1.docx]

**Supplementary Material 1: Database Search Strategy**

Terms were searched in all fields, specifically relevant fields included title, abstract and body of text.

Table 1: Search strategy used for Embase via Ovid, last searched 9th May 2024

| **#** | **Search Term** | **Boolean Operator** | **Search Result** |
| --- | --- | --- | --- |
| 1 | exp Medication Systems, Hospital/ or automated dispensing.mp. | OR | 3779 |
| 2 | automated dispensing.mp. | OR | 289 |
| 3 | exp Medication Systems/ | OR | 6217 |
| 4 | implementation.mp. |  | 383741 |
| 5 | automated medication.mp. |  | 122 |
| 6 | medication system.mp. |  | 161 |
| 7 | 2 or 5 |  | 399 |
| 8 | 3 or 6 |  | 6312 |
| 9 | 1 and 4 and 7 and 8 |  | 38 |
| 10 | automated dispensing cabinet.mp. |  | 54 |
| 11 | 9 or 10 |  | 86 |
| 12 | limit 11 to (english language and full text) |  | 14 |

Table 2: Search strategy used for PubMed, last searched 9th May 2024

| **#** | **Search Term** | **Boolean Operator** | **Search Result** |
| --- | --- | --- | --- |
| 1 | ((automated medication) OR (automated dispensing)) OR (automated distribution) | OR | 90,038 |
| 2 | (hospital) OR (inpatient) | OR | 7,256,070 |
| 3 | automated dispensing cabinet |  | 155 |
| 4 | (((implementation) OR (outcome)) OR (advantage)) OR (disadvantage) | OR | 4,247,252 |
| 5 | 2 AND 3 |  | 140 |
| 6 | 2 AND 3 AND 4 |  | 72 |
| 7 | 1 AND 7 |  | 72 |
| 8 | limit 7 to (english language) |  | 66 |

Table 3: Search strategy used for CINAHL, last searched 9th May 2024

| **#** | **Search Term** | **Boolean Operator** | **Search Result** |
| --- | --- | --- | --- |
| 1 | automated medications dispensing system |  | 15 |
| 2 | exp hospital |  | 2,007,176 |
| 3 | exp implementation |  | 177,084 |
| 4 | automated dispensing cabinet |  | 37 |
| 5 | 1 OR 4 |  | 52 |
| 6 | 2 AND 5 |  | 18 |
| 7 | 6 AND 3 |  | 7 |
| 8 | limit to (english language) |  | 7 |

Table 4: Search strategy used for Global Health, last searched 9th May 2024

| **#** | **Search Term** | **Boolean Operator** | **Search Result** |
| --- | --- | --- | --- |
| 1 | automated dispensing cabinet.mp. |  | 5 |
| 2 | automated medication.mp. |  | 6 |
| 3 | exp usage/ |  | 7123 |
| 4 | hospital.mp. |  | 312283 |
| 5 | 1 OR 2 |  | 11 |
| 6 | 4 AND 5 |  | 5 |
| 7 | limit 6 to (english language) |  | 4 |

Table 5: Search strategy used for Web of Science, last searched 10th May 2024

| **#** | **Search Term** | **Boolean Operator** | **Search Result** |
| --- | --- | --- | --- |
| 1 | automated drug dispensing system OR automated dispensing cabinet OR automated drug dispensing system OR automated drug dispensing |  | 542 |
| 2 | exp hospital* |  | 2043756 |
| 3 | exp usage OR exp implementation | OR | 525743 |
| 4 | 1 AND 2 AND 3 |  | 92 |
| 5 | limit 4 to (english language) |  | 78 |
